# Heterogeneity Primer Spacers Improve the Performance of Massively Parallel Amplicon Sequencing of the V3-V4 Region of the 16 S rDNA as well as the 18 S Region for *Blastocystis* Subtyping

**DOI:** 10.1007/s00248-026-02708-3

**Published:** 2026-03-04

**Authors:** Ondrej Cinek, Klára Hubáčková, Karolína Litošová, Lucie Hlináková

**Affiliations:** Department of Medical Microbiology, Second Faculty of Medicine, Charles University, University Hospital Motol, V Úvalu 84, Prague 5, Prague, Czechia CZ-15006 Czechia

**Keywords:** Amplicon profiling, Heterogeneity, Staggering, Bacteriome

## Abstract

The Illumina MiSeq and NextSeq instruments are workhorses for many low- to medium-throughput amplicon sequencing applications in microbiology; however, their performance is highly sensitive to decreased diversity of libraries. Here, we present a systematic, algorithm-driven approach to designing heterogeneity spacers for amplification primers and demonstrate their use in two marker gene profiling applications. Heterogeneity spacers were designed to balance the sequencing signal, ensuring that no sequencing cycle contained more than 60% of a single dominant base. Additional bases were inserted into the first-round amplification primer adjacent to the target-specific portion. A simple script was written to remove the heterogeneity spacers from the reads, and verify equal representation of primer combinations. We designed sixteen staggered primer variants for profiling the V3-V4 regions of the 16S bacterial rDNA gene and sixteen for bidirectional profiling of the 18S rDNA region informative for *Blastocystis* subtypes. The effectiveness of our design was evident from the increased heterogeneity of the sequencing signal, with no primer combination predominating. The method was tested against classical primer design and has successfully been applied to several thousand samples to date. Inserting heterogeneity spacers before the target-specific portion of the first-round PCR primers is a simple and reliable way to increase the diversity of marker gene amplicons. The multiplexed reactions produce amplicons that can be easily sequenced on Illumina platforms, eliminating the need for high concentrations of balancing PhiX library.

**Supplementary Information:**

The online version contains supplementary material available at 10.1007/s00248-026-02708-3.

Massive parallel sequencing of PCR amplicons is widely used for various applications in molecular microbiology - primarily for bacteriome profiling using the 16S rDNA gene, but also for molecular subtyping in virology and parasitology. All of these applications rely on primers that anneal to highly conserved gene regions flanking informative polymorphic sequences, the variability of which allows individual reads to be classified into taxa or subtypes. The high capacity, relatively straightforward amplicon library indexing and clean-up protocols, and reasonable costs have made the Illumina MiSeq and, more recently, the NextSeq sequencers the workhorses of these applications. Both are capable of deep sequencing of 2 × 300 bases, meaning the amplicon of interest can be up to 550–580 bases long, with the paired-end reads overlapping and able to be joined into a single longer sequence.

However, this instrumentation suffers from a considerable drawback for amplicon sequencing: the sequencers fail to sequence signals that are homogeneous across the flow cell. This means that, in any given sequencing cycle, multiple differently fluorophore-coded bases must always be present at a sufficient proportion and, ideally, at a balanced ratio. If this is not the case - especially at the start of the sequencing run - the clusters (spots of clonal origin on the flow cell) are not correctly identified, the colour matrix corrections are not established, the sequencing quality dramatically decreases, potentially causing the sequencing run to fail [1]. The need for balanced signal applies to both the insert (amplicon) reads, and the reads of indices that identify the samples. In the index reads, colour balancing is only a problem in low-plexity runs, and there are easy-to-follow rules for which indices should be combined [2, 3]. The situation is more problematic with the colour balance of sequenced PCR amplicons. As these start with conserved primers and usually contain highly conserved regions, their base content must be balanced by adding a heterogeneous metagenomic control library derived from the *PhiX* bacteriophage (PhiX Control Library, Illumina). The recommended proportion of the *PhiX* library can be rather high; for example, in a NextSeq 1000/2000 sequencing run of a low diversity library, the addition of 40% is recommended, alongside a reduction in the loading concentration to 30–40% below the optimal cluster density [4, 5]. Together, these two steps inevitably lead to a dramatic loss in sequencing capacity.

To avoid wasting sequencing capacity and reduce the risk of failures, primer staggering (phasing) has been introduced as an alternative. This strategy uses multiple variants of amplification primers that have a variable number of extra bases (heterogeneity spacers) inserted *before* the specific portion, resulting in amplicons that are offset from each other. Such offsets make the sequencing signal heterogeneous. There are many possible strategies for base insertions, which vary considerably in terms of set-up costs, the workload involved in wet lab procedures, and the need for additional bioinformatic analysis steps [6–11].

Our objective was to develop a simple primer staggering method to increase amplicon library diversity and enhance the performance of massively parallel amplicon sequencing on the Illumina platform. We achieved this in 16S V3-V4 rDNA profiling (an example of an amplicon with hundreds of bacterial sequence variants per sample) and in *Blastocystis* sp. subtyping (an example of an amplicon with one or two subtypes per sample).

To increase the diversity of amplicons, we (a) added staggering (heterogeneity spacers) to existing primers for the amplification of the 16 S rDNA gene (regions V3-V4), and for a subtype-informative region of *Blastocystis* sp., and (b) designed amplicons in both direct and inverted orientations. This design was developed in several iterations: from a previous sequencing run without staggering, we obtained and rarefied data of several typical samples. Using an R script, we simulated the addition of heterogeneity spacers, and sequencing in direct and reverse orientation together. We plotted and inspected the base proportions along the length of the sequencing reads, with the goal of achieving no more than 60% predominance of any base anywhere along the read. The final design was the result of a iterative process when the user tried different variants of heterogeneity spacers while observing the effects on the evenness of the simulated signal. The script used to simulate the staggering along with the relevant test data have been deposited at https://github.com/ondrejcinek/primer_spacers/DESIGN_staggered_primers.Rmd, and can be directly run at https://ondrejcinek.shinyapps.io/design_staggered_primers_app/ when the usage limits allow it.

The heterogeneity spacers are used in the first round of PCR; therefore, the subsequent steps (clean-up, indexing, clean-up with normalisation and sequencing) follow standard Illumina protocols. The location of the spacers and an outline of the workflow is shown in Fig. 1. For details on the procedure refer to Supplementary Materials, Protocol 1. Once sequencing is complete, the sequences of heterogeneity spacers are removed and the orientation of the reads is unified using a simple Python script deposited at https://github.com/ondrejcinek/primer_spacers/process_staggered_run.py.

**Fig. 1:**
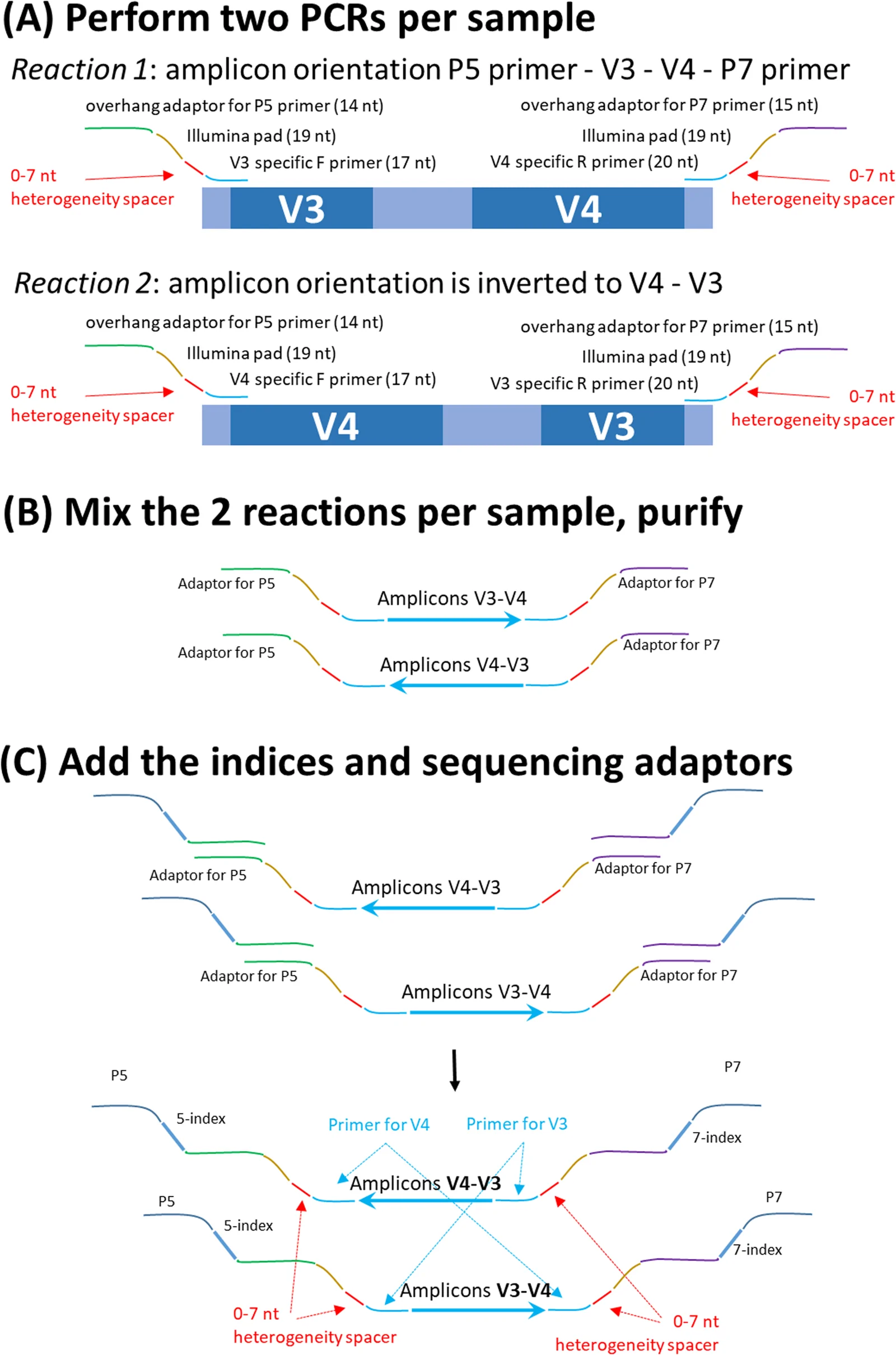
**The localisation of the heterogeneity spacers and the workflow exemplified on the 16S rDNA profiling**

The resulting primers are shown in Table 1. These staggered primers have been used in several thousand bacteriome profiling reactions and several hundred *Blastocystis* subtyping reactions on DNA extracted from faecal material by the DNeasy PowerSoil Pro kit (Qiagen, Hilden, Germany): e.g. 16S rDNA profiling studies submitted to NCBI Sequence Read Archive under projects PRJNA1273891, PRJNA1367098, or *Blastocystis* subtyping study under PRJNA1121083. Two separate PCR reactions are set up for each sample, one for the direct orientation of the amplicon relative to the sequencing adaptors P5 and P7, and the other for the reverse orientation of the amplicon. Each reaction contains eight primer variants. For 16S rDNA amplicons the total number of bases added ranges from 0 to 11 for the direct orientation and from 0 to 13 for the reverse orientation. These additions still permit the use of 2 × 250 MiSeq v2 sequencing kits for 16S rDNA, since the total length of the bacterial amplicons enables a sufficiently long overlap of the Illumina reads. In *Blastocystis* subtyping this range is 0–6, but the longer amplicon requires sequencing at 2 × 275 bases or more, thus requiring the MiSeq or NextSeq sequencing kits with a capacity of 2 × 300. Figure 2 compares the quality score charts (panel A) and base proportion (panels B and C) between a typical run before the introduction of primer staggering, and a run with staggered primers.

**Fig. 2:**
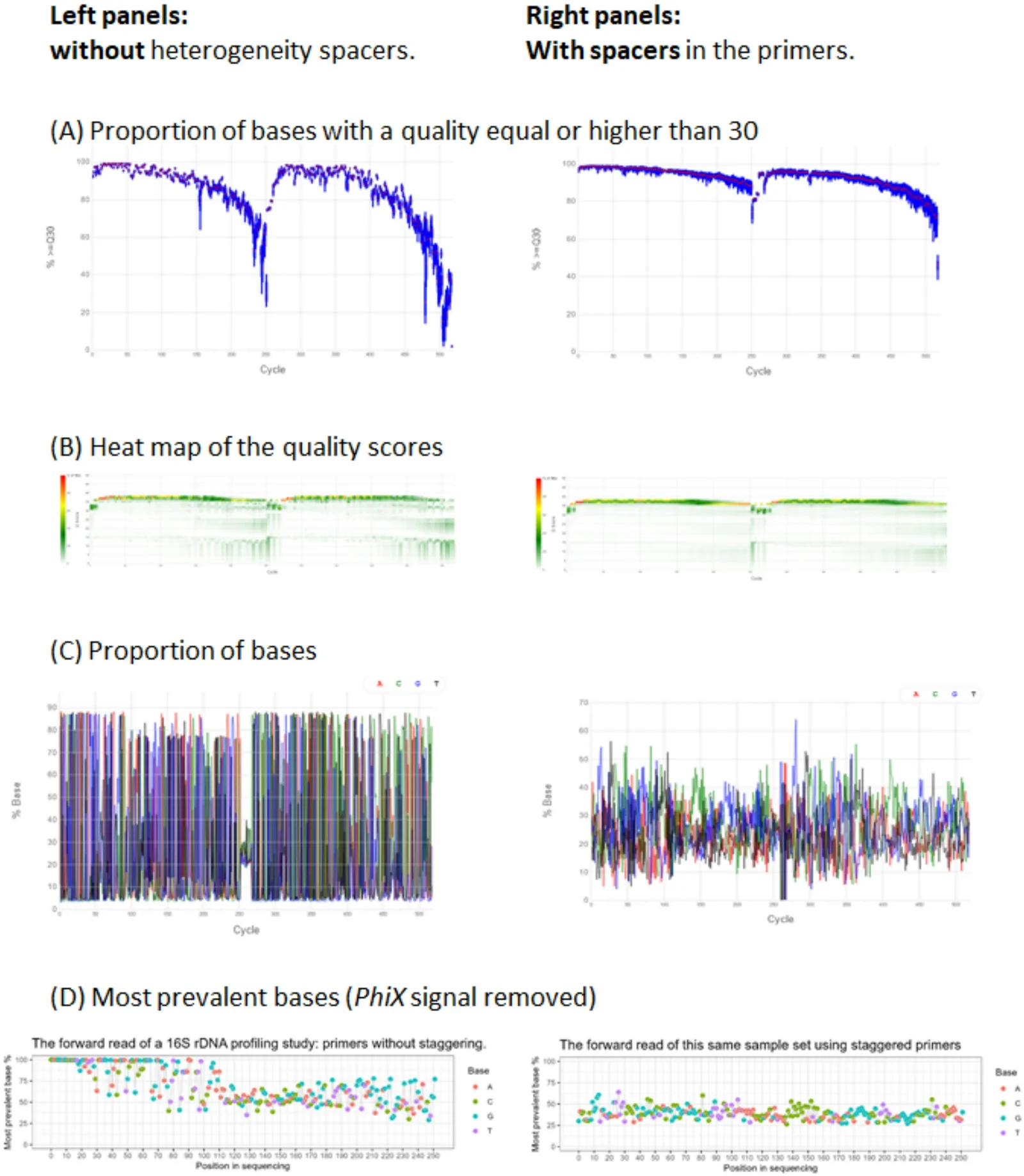
**Comparison of two sequencing runs: one without and one with the novel staggered primers. **The left panel shows data from a sequencing run according to Kozich et al. [16], with an addition of 20% *PhiX* library (the lowest concentration that allowed completing of the run without sequencing failure). The right panel shows the result of another sequencing run performed according to the presently developed protocol with staggered primers, using a 10% addition of the *PhiX* library. Panels A-C are from Illumina BaseSpace on-line database, whereas plots in panel C are generated by our scripts. Improvement in quality scores are shown in Panels A and B, whereas panels C and D document that heterogeneity spacers help eliminate the predominance of single bases

**Table 1:** Sequences of the staggered primers used for the first round of PCR

(A) Primers for profiling the V3-V4 region of the *16S rDNA*
**Reaction 1 (direct orientation of the amplicon)**
The reaction 1 Read 1 primers: P5-tag, pad, spacer varying in length, and V3 forward primer
TCGTCGGCAGCGTC*AGATGTGTATAAGAGACAG* **CCTACGGGAGGCAGCAG**
TCGTCGGCAGCGTC*AGATGTGTATAAGAGACAG*ga**CCTACGGGAGGCAGCAG**
TCGTCGGCAGCGTC*AGATGTGTATAAGAGACAG*tag**CCTACGGGAGGCAGCAG**
TCGTCGGCAGCGTC*AGATGTGTATAAGAGACAG*agcaatt**CCTACGGGAGGCAGCAG**
The reaction 1 Read 2 primers: P7-tag, pad, spacer varying in length, and V4 reverse primer
GTCTCGTGGGCTCGG*AGATGTGTATAAGAGACAG* **GGACTACHVGGGTWTCTAAT**
GTCTCGTGGGCTCGG*AGATGTGTATAAGAGACAG*ca.**GGACTACHVGGGTWTCTAAT**
GTCTCGTGGGCTCGG*AGATGTGTATAAGAGACAG*tct**GGACTACHVGGGTWTCTAAT**
GTCTCGTGGGCTCGG*AGATGTGTATAAGAGACAG*atct**GGACTACHVGGGTWTCTAAT**
**Reaction 2 (reverse orientation of the amplicon)**
The reaction 2 Read 1 primers: P5-tag, pad, spacer varying in length, and V4 reverse primer
TCGTCGGCAGCGTC*AGATGTGTATAAGAGACAG* **GGACTACHVGGGTWTCTAAT**
TCGTCGGCAGCGTC*AGATGTGTATAAGAGACAG*ca.**GGACTACHVGGGTWTCTAAT**
TCGTCGGCAGCGTC*AGATGTGTATAAGAGACAG*atct**GGACTACHVGGGTWTCTAAT**
TCGTCGGCAGCGTC*AGATGTGTATAAGAGACAG*tctact**GGACTACHVGGGTWTCTAAT**
The reaction 2 Read 2 primers: P7-tag, pad, spacer varying in length, and V3 forward primer
GTCTCGTGGGCTCGG*AGATGTGTATAAGAGACAG* **CCTACGGGAGGCAGCAG**
GTCTCGTGGGCTCGG*AGATGTGTATAAGAGACAG*ga**CCTACGGGAGGCAGCAG**
GTCTCGTGGGCTCGG*AGATGTGTATAAGAGACAG*tag**CCTACGGGAGGCAGCAG**
GTCTCGTGGGCTCGG*AGATGTGTATAAGAGACAG*agcaatt**CCTACGGGAGGCAGCAG**
**(B) Primers for subtyping of** ***Blastocystis*** **sp.**
**Reaction 1 (direct orientation of the amplicon)**
The reaction 1 Read 1 primers: P5-tag, pad, spacer varying in length, and *Blastocystis* forward primer
TCGTCGGCAGCGTC*AGATGTGTATAAGAGACAG* **GGAGGTAGTGACAATAAATC**
TCGTCGGCAGCGTC*AGATGTGTATAAGAGACAG*a**GGAGGTAGTGACAATAAATC**
TCGTCGGCAGCGTC*AGATGTGTATAAGAGACAG*ca.**GGAGGTAGTGACAATAAATC**
TCGTCGGCAGCGTC*AGATGTGTATAAGAGACAG*act**GGAGGTAGTGACAATAAATC**
The reaction 1 Read 2 primers: P7-tag, pad, spacer varying in length, and *Blastocystis* reverse primer
GTCTCGTGGGCTCGG*AGATGTGTATAAGAGACAG* **TGCTTTCGCACTTGTTCATC**
GTCTCGTGGGCTCGG*AGATGTGTATAAGAGACAG*a**TGCTTTCGCACTTGTTCATC**
GTCTCGTGGGCTCGG*AGATGTGTATAAGAGACAG*ca.**TGCTTTCGCACTTGTTCATC**
GTCTCGTGGGCTCGG*AGATGTGTATAAGAGACAG*act**TGCTTTCGCACTTGTTCATC**
**Reaction 2 (reverse orientation of the amplicon)**
The reaction 2 Read 1 primers: P5-tag, pad, spacer varying in length, and *Blastocystis* reverse primer
TCGTCGGCAGCGTC*AGATGTGTATAAGAGACAG* **TGCTTTCGCACTTGTTCATC**
TCGTCGGCAGCGTC*AGATGTGTATAAGAGACAG*a**TGCTTTCGCACTTGTTCATC**
TCGTCGGCAGCGTC*AGATGTGTATAAGAGACAG*ca.**TGCTTTCGCACTTGTTCATC**
TCGTCGGCAGCGTC*AGATGTGTATAAGAGACAG*act**TGCTTTCGCACTTGTTCATC**
The reaction 2 Read 2 primers: P7-tag, pad, spacer varying in length, and *Blastocystis* forward primer
GTCTCGTGGGCTCGG*AGATGTGTATAAGAGACAG* **GGAGGTAGTGACAATAAATC**
GTCTCGTGGGCTCGG*AGATGTGTATAAGAGACAG*a**GGAGGTAGTGACAATAAATC**
GTCTCGTGGGCTCGG*AGATGTGTATAAGAGACAG*ca.**GGAGGTAGTGACAATAAATC**
GTCTCGTGGGCTCGG*AGATGTGTATAAGAGACAG*act**GGAGGTAGTGACAATAAATC**

Illumina overhang tags: plain uppercase

TCGTCGGCAGCGTC (P5-tag, a Read1 Nextera pre-adapter) and GTCTCGTGGGCTCGG (P7-tag, a Read 2 Nextera pre-adapter). The P5- and P7-tags are the sites where second-round indexing primers anneal.

Illumina pad sequence: underlined. *AGATGTGTATAAGAGACAG*; it is the annealing site of the Illumina sequencing primers. The read 1 and read 2 thus both start from the specific locus primer (or the heterogeneity spacer if it is present). The Illumina overhang tag along with the pad sequence make together the whole overhang sequence - these are the annealing sites for the read 1 sequencing primer (the P5-tag and the following pad) and the read 2 sequencing primer (the P7-tag and the sequencing primer).

Heterogeneity spacers (primer staggering, heterogeneity spacers): ga, tag... (lowercase) Specific locus primers:  in bold

16S rDNA profiling: **CCTACGGGAGGCAGCAG, GGACTACHVGGGTWTCTAAT **(bold uppercase): specific portions of primers V3-forward (341-F [12]) and V4-reverse (806-R [13]).

*Blastocystis *subtyping:** GGAGGTAGTGACAATAAATC, TGCTTTCGCACTTGTTCATC** are specific portions of F (ILMN_Blast505_532F) and R (ILMN_Blast998_1017R) primers for subtyping of *Blastocystis *sp. according to Maloney et al [14]. It is worth noting that these primers also amplify a considerable number of bacterial sequences when used with stool DNA, which increases the diversity of the amplicons.

A detailed comparison of our primers with those in previously published papers, and with the commercially available microbiome profiling kit (QIAseq by Qiagen, Hilden, Germany) is shown in Table 2. In brief, our approach is novel in that it uses both direct and inverted orientations of the amplicons, thereby enhancing the diversity of the sequencing signal. It offers a good balance between ease of PCR setup and initial primer synthesis costs. By contrast, some other studies either employed cumbersome wet-lab procedures, complicated alterations to sequencer configuration files, unpublished non-standard bioinformatic procedures, or incurred prohibitive costs by designing excessive numbers or lengths of primers. We also believe that our publicly available script, which assists with designing the heterogeneity spacers, could help others with the step-by-step creation of spacers that fit specific amplicons and users’ needs. Finally, our protocol modification only involves the first round of PCR; all subsequent steps (clean-up, indexing etc.) follow standard Illumina protocols. This ensures that the full spectrum of available indices can be used for multiplexing, with the number of samples in a run being limited only by the desired sequencing depth, and the type of sequencing kit. This is not the case in several other protocols where staggering was included in the second-round primers [10], single-round amplification was performed using very long primers [8, 11], or only 96 index combinations were available (QIAseq by Qiagen, Hilden, Germany).

**Table 2:** Comparison to previously published works using heterogeneity spacers in 16S rDNA profiling

	The present work	Jensen et al., 2019 [9]	Wu et al., 2015 [10]	Fadrosh et al., 2014 [8]	Lundberg et al., 2013 [7]	Gloor et al., 2010 [11]	The QIAseq kit by Qiagen
**Sequenced region**	V3 + V4:V3-forward (341-F, [12]) and V4-reverse (806-*R*, [13])	V3 + V4:338 F [15],806R [13])	V4 (515 F + 806R). Primers have no overhangs.	469 bp of V3 + V4 (319 F − 806R)	V4 (515–806 of E. coli)	V6 (primers at 967–985 and 1078–1061 of E.coli)	(a) either all these amplicons: V1V2, V2V3, V3V4, V4V5, V5V7, V7V9, ITS (“screening panel”), or (b) specific selected region (“region panel”)
**Primers**	The first round has 2 reactions with 8 primers each. The second round is a standard indexing amplification with up to 384 combinations.	First-round primers modified by an addition of 0–7 bases after the specific part.	Preamplification by primers without tails. Second-round primers target the specific amplicon only. The reverse primer is barcoded.	Single round of amplification. Spacers 0–7 bases inserted between the specific and index portion.	Tagging primers contain the specific portion, link, 5–10 bases of the molecular tag, and an overhang for the amplification primer. Amplification primers contain Read 1 and Read 2 adapters for linking to the sequencing flow-cell.	5’ - end of the primer extended by varying length of tags (3–6 nt). 18 tags in left primer 16 tags in right primer	Proprietary; 3 reactions.
**Heterogeneity spacers**	0–7 bases inserted in the first-round primers	0–7 bases inserted in the first-round primers	In second-round primers, a total addition of 7 bases in all combinations	Single-round amplification primers, 0–7 bases inserted	The insert serves as a molecular tag, 5–10 bases.	Single-round PCR, the spacers 3–6 nt serve also as a barcode	0–11 bases inserted next to the specific primer
**Wet lab workflow**	A standard Illumina nested amplicon PCR: the first round (2 reactions per sample), Clean-up (1x) of both reactions mixed. Second round of PCR for indexing Clean-up, Library QC Pooling + sequencing	Very complicated, with 10 separate reactions per sample in the first PCR round.	Very easy: first round of PCR with normal primers second round of PCR with predefined primer mixes.	Very easy: A single round of PCR; Clean-up; Pooling and sequencing	Very complicated: reverse tagging + clean-up using beads; forward tagging + clean-up using beads; PCR with reverse primers barcoded; clean-up using beads; library QC, pooling + sequencing with a custom primer	Single round of PCR - the spacers serve also for barcoding.	First round of PCR (3 reactions/sample), 2x clean-up, second round for indexing, Clean-up, Library QC, Pooling + Sequencing A kit with detailed instructions.
**Sequencing**	2 × 250 or more bases for 16 S rDNA, and 2 × 275 or more for *Blastocystis*	2 × 300 bases		2 × 250 or more	2 × 250 with a custom primer	2 × 75 bp	2 × 300 bases
**Bioinformatic workflow**	Spacer removal + statistics by an open-source script. Any standard workflow can follow.	Trimming of the spacers was not described. Scripts not available. Commercial CLC workbench needed.	Quite complicated, including an alteration to the MiSeq configuration file.	Not available. Scripts have been removed from *github* after publication.	Supported with a software toolbox (freely downloadable at https://github.com/islandhopper81/mt-toolbox). The homepage with instructions etc. is no longer available.	Custom scripts in C and Perl - not publicly available	Qiagen Bioinformatics CLC genomic workbench + Microbial Genomics Pro Suite Module required.
**Availability of bioinformatic tool**	Open-source, deposited on *github*.	Not available.		Not available	Instructions & manuals no longer available.	The code was not published nor deposited.	From Qiagen
**Costs**	Set-up costs are moderate, 16 NGS-grade primers for the first round.	Set-up costs moderate (primers for 10 combinations), then a costly first round of PCR.	Prohibitive costs of single-barcoded primers (over a hundred very long primers have to be synthesized).	Set-up costs rather high, the long single-round primers contain indices.	Not applicable, surpassed by simpler protocols.	Not applicable, surpassed by simpler protocols.	A commercial kit + fees for the above bioinformatic tools
**Strengths**	Adds variance by amplification in a direct and an inverted reaction. The trimming script checks for proportionality of primer combinations.	Primer sequences well documented. Results of the test runs compared.		Simplicity of the single-amplification approach.	This was the first paper with detailed protocols and software publicly available.	One of the pioneering proof-of-principle works (published 2010).	Utilises low bioburden reagents (background is reduced)
**Limitations**	Relies on previously documented evenness of the multiplexed reactions.	Very costly (10 reactions per sample in the first PCR round). Limited heterogeneity of primer combinations. Software not published.	As only the reverse primer is barcoded, primers are specific for the given target, and the second-round primers are long, the set-up costs for primer synthesis are prohibitive.	Scripts no longer available. Spacers paired with indices = possible source of bias.	Excessively laborious wet lab procedure, now surpassed by simpler protocols. The webpage with software instructions is no longer available.	Only V9 region used (short reads - one of the limitations of the technology at the time). Lack of bioinformatic support for the spacer removal.	Maximum multiplexing: 96 samples. Bioinformatic analysis on a proprietary platform only. Total costs.

In conclusion, we provide a simple, well-documented protocol for massively parallel amplicon sequencing on the Illumina platform using staggered primers that artificially increase the heterogeneity of the amplicons, thereby improving the quality of the sequencing data. 

## Supplementary Information

Below is the link to the electronic supplementary material.

Supplementary Material 1 (PDF 1.19 MB)

## Data Availability

No datasets were generated or analysed during the current study.

## Declarations

### Code Repository

The code used to create staggered primers described in this paper is available at https://github.com/ondrejcinek/primer_spacers along with the relevant test data. The application for drawing base frequencies over the NGS reads has also been posted at https://ondrejcinek.shinyapps.io/design_staggered_primers_app/. It can be run there, provided that the free tier’s machine time limit allows it.
