# Supplementary material for: Heterogeneity Primer Spacers Improve the Performance of Massively Parallel Amplicon Sequencing of the V3-V4 Region of the 16 S rDNA as well as the 18 S Region for Blastocystis Subtyping
Source: Microb Ecol. 2026 Mar 4;89(1):76. doi: 10.1007/s00248-026-02708-3 (PMC13009126; doi:10.1007/s00248-026-02708-3)
Supplement: Supplementary file 1 — Supplementary Material 1 (PDF 1.19 MB) [file 248_2026_2708_MOESM1_ESM.pdf]

Massively parallel sequencing of staggered amplicons for 16S rDNA profiling and *Blastocystis* subtyping - the protocol

Supplementary material to the paper Cinek O et al, *Heterogeneity primer spacers improve the performance of massively parallel amplicon sequencing of the V3-V4 region of the 16S rDNA as well as the 18S region for Blastocystis subtyping.*

TABLE OF CONTENTS:

1 Wet lab procedures 2
1.1 First round of the PCR 2
1.1.1 The primer mixes 2
1.1.2 Outline of the procedure 3
1.1.3 Chemicals for the PCR 3
1.1.4 Worksheet for the bacteriome profiling 4
1.1.5 Worksheet for the amplification of *Blastocystis* subtyping fragment 5
1.1.6 Amplification layout worksheet 6
1.2 Merging the two reactions by sample 7
1.3 Indexing, purification, equalisation, sequencing 7
2 Bioinformatics 8
2.1 Removal of primer spacers and statistics of their representation 8
2.2 Optional: merging of the left and right reading of the amplicon 10
3 Annex - list of primers 11
3.1 Primers for bacteriome profiling 11
3.2 Primers for *Blastocystis* subtyping 12

1 WET LAB PROCEDURES

1.1 FIRST ROUND OF THE PCR

1.1.1 THE PRIMER MIXES

The primer sequences are listed in **Table 1** of the manuscript as well as in the Annex to this document.

The **equimolar primer mixtures @20 µM** are prepared from 100 µM primer stocks as follows:
In a DNA-free labelled screw-cap tube mix:

- 320 µl PCR grade water
- a total of 80 µl primer stocks (100 µM), divided among the members of the pool (i.e. for 8 members it is 8 x 10µl)
- prepare multiple such tubes, and freeze

Primer mixes for the bacteriome profiling using 16S rDNA amplification of regions V3 and V4

Prepare a primer mix for reaction 1 named `reaction1_direct` **(V34)** as follows:

- 320 µl PCR grade water
- 10 µl of each primer (100 µM stock):
  Bact341_0_for5, Bact341_2_for5, Bact341_3_for5, Bact341_6_for5, Bact806_0_rev7, Bact806_2_rev7, Bact806_3_rev7, Bact806_4_rev7

Prepare a primer mix pro reaction 2 named `reaction2_reversed` **(V43)** as follows:

- 320 µl PCR grade water
- 10 µl of each primer (100 µM stock):
  B806_341_0_for5, B806_341_2_for5, B806_341_3_for5, B806_341_6_for5, B806_341_0_rev7, B806_341_2_rev7, B806_341_3_rev7, B806_341_6_rev7

Primer mixes for subtyping of *Blastocystis*

Prepare a primer mix for reaction 1 named `reaction1_direct` **(BT_THERE)** as follows:

- 320 µl PCR grade water
- 10 µl of each primer (100 µM stock):
  BT_F0_for5, BT_F1_for5, BT_F2_for5, BT_F3_for5, BT_R0_rev7, BT_R1_rev7, BT_R2_rev7, BT_R3_rev7

Prepare a primer mix pro reaction 2 named `reaction2_reversed` **(BT_BACK)** as follows:

- 320 µl PCR grade water
- 10 µl of each primer (100 µM stock):
  BT_R0_for5, BT_R1_for5, BT_R2_for5, BT_R3_for5, BT_F0_rev7, BT_F1_rev7, BT_F2_rev7, BT_F3_rev7

1.1.2 OUTLINE OF THE PROCEDURE

(1) PCR amplification of the target

- the targets are amplified using the above primer mixes, the product is checked on gel electrophoresis

(2) Merging the two PCR products for each sample

- products of amplification are pooled by sample (i.e. products of different orientations amplified from the same sample will be pooled in one well)

(3) Size-limiting PCR clean-up

- the PCR products are purified of the unused primers and dNTP
- the clean-up also limits the minimum retained size of the product

(4) Index PCR (to attach indices and sequencing adaptors)

- indices and anchor adaptors for the Illumina sequencing are added

(5) Library quantification, normalization, pooling and sequencing

- this is done using normal workflow as for any other library for the MiSeq or NextSeq machine.

1.1.3 CHEMICALS FOR THE PCR

- Phusion Plus DNA polymerase (Thermo Scientific), cat. no. F630 S / L / XL.
- dNTP mix 10 mM each (Thermo Scientific), e.g. cat. no. 18427089
- PCR water - any suitable
- primers: synthesised as NGS-grade (e.g. by Eurofins Genomics)
- clean-up: SPRIselect Beads (Beckman Coulter), cat. no. B23317, B23318, B23319

16S rDNA bacteriome profiling, first PCR round

Name of the run:__________________ **Done on date:** ___________ **By whom**: ___________

A mix for `reaction1_direct` `(V34)`

Component	µl / r.	Master mix µl	First open / Lot #	remarks
**PCR water**	11,4		-	
**5x buffer**	4,0			
**dNTP** (Thermo) 10 mM	0,4			
**reaction1_direct (V34)**	2,0			
**Phusion Plus DNA Pol.**	0,2			
**DNA template**	2,0		-	
Total	20 µl		-	

A mix for `reaction2_reversed` `(V43)`

Component	µl / r.	Master mix µl	First open / Lot #	remarks
**PCR water**	11,4		-	
**5x buffer**	4,0			
**dNTP** (Thermo) 10 mM	0,4			
**reaction2_reversed (V43)**	2,0			
**Phusion Plus DNA Pol.**	0,2			
**DNA template**	2,0		-	
Total	20 µl		-	

Run the PCR program. Use slow ramp speeds (<= 1°C/sec.).

**PCR program**	**"Phusion Plus"**
**initial denat.**	98°C, 0:30
**cycling**	**20x / 30x / 40x** [1]
	98°C, 0:10
	60°C, 0:30
	72°C, 0:30
**finally**	72°C, 5:00
	10°C, infinity

Thermocycler: date and time:_________________; name of cycler ____________________

Electrophoresis done? yes / no; by whom ___________________
Do not mix any dyes into the PCR product! Will be used for the downstream protocol.

[1] Depends on the quantified amount of bacterial genomes in the sample. Remember that this PCR with tailed primers needs more cycles compared to the real-time PCR detection & quantitation assay of 16S rDNA.

Blastocystis subtyping, first PCR round

Name of the run:__________________ **Done on date:** __________ **By whom**: __________

A mix for `reaction1_direct` `(BT_THERE)`

Component	µl / r.	Master mix µl	First open / Lot #	remarks
**PCR water**	11,4		-	
**5x buffer**	4,0			
**dNTP** Thermo 10 mM	0,4			
**reaction1_direct (BT_THERE)**	2,0			
**Phusion Plus DNA Pol.**	0,2			
**DNA template**	2,0		-	
Total	20 µl		-	

A mix for `reaction2_reversed` `(BT_BACK)`

Component	µl / r.	Master mix µl	First open / Lot #	remarks
**PCR water**	11,4		-	
**5x buffer**	4,0			
**dNTP** Thermo 10 mM	0,4			
**reaction2_reversed (BT_BACK)**	2,0			
**Phusion Plus DNA Pol.**	0,2			
**DNA template**	2,0		-	
Total	20 µl		-	

Run the PCR program. Use slow ramp speeds (<= 1°C/sec.).

**PCR program**	**"Phusion Plus"**
**initial denat.**	98°C, 0:30
**cycling**	**30x / 40x / 50x** [2]
	98°C, 0:10
	60°C, 0:30
	72°C, 0:30
**finally**	72°C, 5:00
	10°C, infinity

Thermocycler: date and time:________________; name of cycler ___________________

Electrophoresis done? yes / no; by whom __________________
Do not mix any dyes into the PCR product! Will be used for the downstream protocol.

[2] Depends on the quantified amount of organism in the sample. Remember that this PCR with tailed primers needs more cycles compared to the real-time PCR detection & quantitation assay. Add some 5-10 cycles.

1.1.6 AMPLIFICATION LAYOUT WORKSHEET

Name of the run / PCR plate ____

	1	2	3	4	5	6	7	8	9	10	11	12
A:												
B:												
C:												
D:												
E:												
F:												
G:												
H:												

1.2 MERGING THE TWO REACTIONS BY SAMPLE

Two PCR products have been prepared for each sample and checked on a gel electrophoresis - one with a direct orientation of the amplicon, the other with reverse orientation.

Merge the two reactions 1:1, achieving the volume that will be used for purification. E.g. if the downstream purification will use 20 µl, merge 10 µl + 10 µl.

1.3 INDEXING, PURIFICATION, EQUALISATION, SEQUENCING

The upstream procedure generated amplicons that will be now processed using standard pipeline for Illumina tagged amplicons. This is essentially identical for all kinds of tailed amplicons. Please refer to the document on 16S profiling by Illumina (they call it *metagenomic* sequencing, which is technically not accurate), the document 15044223 Rev. B, http://support.illumina.com/documents/documentation/chemistry_documentation/16s/16s-metagenomic-library-prep-guide-15044223-b.pdf. Find "*alternative amplicon primers*" on page 3 of the above cited document.

The procedure:

- The process starts with **PCR clean-up** of the merged reaction. The procedure is outlined on page 8 of the above document. Now the SPRI-Select is used instead of Ampure, with the advantage of storage at room temperature, and a better definition of size selection ranges. **The ratio of pooled PCR product to SPRI Select is 1 : 0.8 for both targets**

- **Dual indices are then attached by a limited number of PCR cycles**. These can be combinatorial as described on pages 10-12, or unique dual indices combinations can be utilised, purchased directly from Illumina (described here https://support-docs.illumina.com/SHARE/AdapterSequences/Content/SHARE/AdapterSeq/Illumina_DNA/IlluminaUDIndexes.htm). Both types of indices use the primary overhang (P5- or P7-tag) as the priming site

```
the indexed primer for the 5-side (index 2 primer)
5' AATGATACGGCGACCACCGAGATCTACAC[index 5 (8 or 10 bases)]TCGTCGGCAGCGTC
          29 bases           +     8-10  bases           + 14 bases

the indexed primer for the 7-side (index 1 primer)
5' CAAGCAGAAGACGGCATACGAGAT[index 7 (8 or 10 bases)]GTCTCGTGGGCTCGG
          24 bases           +     8-10 bases       + 15 bases
```

- The indexed products are again **purified** (page 13-14), and optionally verified on a Bioanalyser chip.

- The following steps are **pooling and library quality control**, with protocols dependent on the sequencing platform (MiSeq, NextSeq).

- The herein described targets can be sequenced at 2x275 bp or more; the longer overlap can enhance the control over downstream merging of the amplicons. If needed, the 16S rDNA amplicons can be sequenced even at 2x250 bp using the older versions of sequencing kits, but in such a case the merging parameters must be adjusted accordingly (see below).

2 BIOINFORMATICS

Download the demultiplexed fastq files into a dedicated folder. Use several randomly chosen *fastq* files in order to determine whether and where trimming is needed (parameters `-trim_last_bases_r1 and -trim_last_bases_r2` below). Beware of making the reads too short for merging.

2.1 REMOVAL OF PRIMER SPACERS AND STATISTICS OF THEIR REPRESENTATION

The heterogeneity spacers are removed, and basic read count statistics performed using ***process_staggered_run.py*** script.

The script can be downloaded from https://github.com/ondrejcinek/primer_spacers

Run it e.g. by typing `python .\process_staggered_run.py` in a directory where the script is located.

A help can be invoked by -h.

```
>python ./process_staggered_run.py -h

usage: process_staggered_run.py [-h]
             -source_dir SOURCE_DIR
             -target_dir TARGET_DIR
             [-track_tables_fn TRACK_TABLES_FN]
             [-primer_description_file PRIMER_DESCRIPTION_FILE]
             [-usearch USEARCH]
             [-merge_pairs_by_usearch MERGE_PAIRS_BY_USEARCH]
             [-trim_last_bases_r1 TRIM_LAST_BASES_R1]
             [-trim_last_bases_r2 TRIM_LAST_BASES_R2]
             [--trim_only_spacers]
             [--trim_whole_primer]
             [--do_not_trim_whole_primer]
             [--write_to_files_of_combinations]
             [--do_not_overwrite]
             [-fastq_minmergelen FASTQ_MINMERGELEN]
             [-read_length READ_LENGTH]
             [-fastq_maxmergelen FASTQ_MAXMERGELEN]
             [-fastq_maxdiffs FASTQ_MAXDIFFS]
             [-max_reads_per_sample MAX_READS_PER_SAMPLE]

Processes a sequencing run with staggered primers into a trimmed read set. For help type:
python assess_staggered_primers.py -h

options:
  -h, --help
show this help message and exit

  -source_dir SOURCE_DIR
The parent directory containing the fastq / fastq.gz files from the sequencing run
(required)

  -target_dir TARGET_DIR
The target directory for the files of output reads (required)

  -track_tables_fn TRACK_TABLES_FN A name for herein generated tables with representation
of bases in the sequencing files. Not needed unless you test a new primer sets. (not
required)
```

-primer_description_file PRIMER_DESCRIPTION_FILE A primer description file formatted as specified in the documentation (not required; default = primers_16SV34_staggered.txt, for 16S rDNA).

-usearch USEARCH
Full path to the USEARCH program. Will be used for merging the paired reads. If not installed, get one at https://www.drive5.com/usearch/download.html - the free 32-bit version is sufficient

-merge_pairs_by_usearch MERGE_PAIRS_BY_USEARCH
1 / 0 whether we will merge the left and right reads by usearch... default to 0

-trim_last_bases_r1 TRIM_LAST_BASES_R1
the read 1 - how many bases should be trimmed from the end? Some MiSeq runs have the last base of very low quality. default = 0

-trim_last_bases_r2 TRIM_LAST_BASES_R2
the read 2 - how many bases should be trimmed from the end? Some MiSeq runs have the last base of very low quality. default = 0

--trim_only_spacers
What to trim - only the heterogeneity spacers, whereas primers are retained? Default = False

--trim_whole_primer
What to trim - trim the whole primers? default = True

--do_not_trim_whole_primer
What to trim - leave the primers on the amplicon? Overrides the above default option.

--write_to_files_of_combinations
Should fastq files be generated also for all single combination of primers? default = False

--do_not_overwrite
Continue where (if) the previous process stopped? default = False

-fastq_minmergelen FASTQ_MINMERGELEN
minimum length of the merged sequence. Default 350 (because of V3-4 of the 16S rDNA; a bit too short for Blastocystis subtyping)

-read_length READ_LENGTH
The read length in this sequencing. Default 300

-fastq_maxmergelen FASTQ_MAXMERGELEN
maximum length of the merged sequence. Default 500 (works well both for the V3-4 of the 16S rDNA, and Blastocystis subtyping)

-fastq_maxdiffs FASTQ_MAXDIFFS
maximum differences between the merged pair memberss . Default 5 (increase for longer overlaps)

-max_reads_per_sample MAX_READS_PER_SAMPLE
Maximum reads retained per sample. If not given, or 0, all reads will be written to output. Otherwise the given number will be randomly chosen.

Note: the reads do not have to be merged - but if the user decides for merging the left and right reads, then the USEARCH program should be installed, and its path specified as shown above.

2.2 OPTIONAL: MERGING OF THE LEFT AND RIGHT READING OF THE AMPLICON

16S rDNA profiling: the amplicon can be sequenced at 2x250 bp - but these relatively short reads require adjusting the of the merging procedure. The overlap of the 16S rDNA reads is between 20 and 34 bases. Therefore, the expected minimum overlap should be set to 15, and the reads should not be trimmed. If the end of the read suffers from poor quality, discard the whole pair.

***Blastocystis* subtyping:** the amplicon should be sequenced at 2x275 bp or more; this provides a wide safety margin for unknown subtypes. Standard parameters for merging can be used.

3 ANNEX - LIST OF PRIMERS

3.1 PRIMERS FOR BACTERIOME PROFILING

Designation	Staggered primers for profiling the V3-V4 region of 16S DNA
	Primers for reaction 1 (direct orientation of the amplicon)
	primer mix V34
Bact341_0_for5	TCGTCGGCAGCGTCAGATGTGTATAAGAGACAGCCTACGGGAGGCAGCAG
Bact341_2_for5	TCGTCGGCAGCGTCAGATGTGTATAAGAGACAGgaCCTACGGGAGGCAGCAG
Bact341_3_for5	TCGTCGGCAGCGTCAGATGTGTATAAGAGACAGtagCCTACGGGAGGCAGCAG
Bact341_6_for5	TCGTCGGCAGCGTCAGATGTGTATAAGAGACAGagcaattCCTACGGGAGGCAGCAG
Bact806_0_rev7	GTCTCGTGGGCTCGGAGATGTGTATAAGAGACAGGGACTACHVGGGTWTCTAAT
Bact806_2_rev7	GTCTCGTGGGCTCGGAGATGTGTATAAGAGACAGcaGGACTACHVGGGTWTCTAAT
Bact806_3_rev7	GTCTCGTGGGCTCGGAGATGTGTATAAGAGACAGtctGGACTACHVGGGTWTCTAAT
Bact806_4_rev7	GTCTCGTGGGCTCGGAGATGTGTATAAGAGACAGatctGGACTACHVGGGTWTCTAAT
	Primers for reaction 2 (reverse orientation of the amplicon)
	primer mix V43
B806_341_0_for5	TCGTCGGCAGCGTCAGATGTGTATAAGAGACAGGGACTACHVGGGTWTCTAAT
B806_341_2_for5	TCGTCGGCAGCGTCAGATGTGTATAAGAGACAGcaGGACTACHVGGGTWTCTAAT
B806_341_3_for5	TCGTCGGCAGCGTCAGATGTGTATAAGAGACAGatctGGACTACHVGGGTWTCTAAT
B806_341_6_for5	TCGTCGGCAGCGTCAGATGTGTATAAGAGACAGtctactGGACTACHVGGGTWTCTAAT
B806_341_0_rev7	GTCTCGTGGGCTCGGAGATGTGTATAAGAGACAGCCTACGGGAGGCAGCAG
B806_341_2_rev7	GTCTCGTGGGCTCGGAGATGTGTATAAGAGACAGgaCCTACGGGAGGCAGCAG
B806_341_3_rev7	GTCTCGTGGGCTCGGAGATGTGTATAAGAGACAGtagCCTACGGGAGGCAGCAG
B806_341_6_rev7	GTCTCGTGGGCTCGGAGATGTGTATAAGAGACAGagcaattCCTACGGGAGGCAGCAG

3.2 PRIMERS FOR *BLASTOCYSTIS* SUBTYPING

Designation	Staggered primers for subtyping *Blastocystis* sp.
	**Primers for reaction1_(direct orientation of the amplicon)**
	**primer mix BT_THERE**
**BT_F0_for5**	TCGTCGGCAGCGTCAGATGTGTATAAGAGACAGGGAGGTAGTGACAATAAATC
**BT_F1_for5**	TCGTCGGCAGCGTCAGATGTGTATAAGAGACAGaGGAGGTAGTGACAATAAATC
**BT_F2_for5**	TCGTCGGCAGCGTCAGATGTGTATAAGAGACAGcaGGAGGTAGTGACAATAAATC
**BT_F3_for5**	TCGTCGGCAGCGTCAGATGTGTATAAGAGACAGactGGAGGTAGTGACAATAAATC
**BT_R0_rev7**	GTCTCGTGGGCTCGGAGATGTGTATAAGAGACAGTGCTTTCGCACTTGTTCATC
**BT_R1_rev7**	GTCTCGTGGGCTCGGAGATGTGTATAAGAGACAGaTGCTTTCGCACTTGTTCATC
**BT_R2_rev7**	GTCTCGTGGGCTCGGAGATGTGTATAAGAGACAGcaTGCTTTCGCACTTGTTCATC
**BT_R3_rev7**	GTCTCGTGGGCTCGGAGATGTGTATAAGAGACAGactTGCTTTCGCACTTGTTCATC
	**Primers for reaction 2 (reverse orientation of the amplicon)**
	**primer mix BT_BACK**
**BT_R0_for5**	TCGTCGGCAGCGTCAGATGTGTATAAGAGACAGTGCTTTCGCACTTGTTCATC
**BT_R1_for5**	TCGTCGGCAGCGTCAGATGTGTATAAGAGACAGaTGCTTTCGCACTTGTTCATC
**BT_R2_for5**	TCGTCGGCAGCGTCAGATGTGTATAAGAGACAGcaTGCTTTCGCACTTGTTCATC
**BT_R3_for5**	TCGTCGGCAGCGTCAGATGTGTATAAGAGACAGactTGCTTTCGCACTTGTTCATC
**BT_F0_rev7**	GTCTCGTGGGCTCGGAGATGTGTATAAGAGACAGGGAGGTAGTGACAATAAATC
**BT_F1_rev7**	GTCTCGTGGGCTCGGAGATGTGTATAAGAGACAGaGGAGGTAGTGACAATAAATC
**BT_F2_rev7**	GTCTCGTGGGCTCGGAGATGTGTATAAGAGACAGcaGGAGGTAGTGACAATAAATC
**BT_F3_rev7**	GTCTCGTGGGCTCGGAGATGTGTATAAGAGACAGactGGAGGTAGTGACAATAAATC
